# Alleviation of Cadmium Toxicity in Thai Rice Cultivar (PSL2) Using Biofertilizer Containing Indigenous Cadmium-Resistant Microbial Consortia

**DOI:** 10.3390/plants12203651

**Published:** 2023-10-23

**Authors:** Ladda Seang-On, Weeradej Meeinkuirt, Preeyaporn Koedrith

**Affiliations:** 1 Faculty of Environment and Resource Studies, Mahidol University, 999 Phuttamonthon District, Nakhon Pathom 73170, Thailand; 2 Water and Soil Environmental Research Unit, Nakhonsawan Campus, Mahidol University, Nakhonsawan 60130, Thailand; 3 Institute of Environmental Medicine for Green Chemistry, Department of Life Science, Biomedical Campus, Dongguk University, 32, Dongguk-ro, Ilsandong-gu, Goyang-si 410-820, Republic of Korea

**Keywords:** bioremediation, biofertilizer, Cd-resistant microbial consortia, phytotoxicity, indigenous soil microorganisms

## Abstract

Biofertilizer as an amendment has growing awareness. Little attention has been paid to bioremediation potential of indigenous heavy-metal-resistant microbes, especially when isolated from long-term polluted soil, as a bioinoculant in biofertilizers. Biofertilizers are a type of versatile nutrient provider and soil conditioner that is cost-competitive and highly efficient with nondisruptive detoxifying capability. Herein, we investigated the effect of biofertilizers containing indigenous cadmium (Cd)-resistant microbial consortia on rice growth and physiological response. The Thai rice cultivar PSL2 (*Oryza sativa* L.) was grown in Cd-enriched soils amended with 3% biofertilizer. The composition of the biofertilizers’ bacterial community at different taxonomic levels was explored using 16S rRNA gene Illumina MiSeq sequencing. Upon Cd stress, the test biofertilizer had maximum mitigating effects as shown by modulating photosynthetic pigment, MDA and proline content and enzymatic antioxidants, thereby allowing increased shoot and root biomass (46% and 53%, respectively) and reduced grain Cd content, as compared to the control. These phenomena might be attributed to increased soil pH and organic matter, as well as enriched beneficial detoxifiers, i.e., *Bacteroidetes, Firmicutes* and *Proteobacteria*, in the biofertilizers. The test biofertilizer was effective in alleviating Cd stress by improving soil biophysicochemical traits to limit Cd bioavailability, along with adjusting physiological traits such as antioxidative defense. This study first demonstrated that incorporating biofertilizer derived from indigenous Cd-resistant microbes could restrict Cd contents and consequently enhance plant growth and tolerance in polluted soil.

## 1. Introduction

Heavy metal pollution of soils has been a concerning and persistent environmental problem worldwide. Canal irrigation water mixed together with untreated industrial and agricultural wastewater has been considered a major source of soil pollution, thereby degrading agricultural land [1,2]. Releasing large quantities of untreated wastewater containing heavily toxic metals, i.e., cadmium (Cd), arsenic (As), nickel (Ni), lead (Pb) and chromium (Cr), and letting them transfer by irrigation to water bodies and soils have led to toxicity and lower yields of unsafe crops with unsatisfactory quality [3,4]. Cd contamination of soil from various potential sources including mining and smelting, sewage sludge in agriculture and industrial releases constituted a severe environmental issue because Cd is a nonbiodegradable and highly toxic element conferring deleterious impacts on the food chain and human health [5,6]. It exhibited extreme toxicity even at low concentrations, owing to its high accumulation, mobility and persistency in living systems [7]. Cellular Cd influx in plants was mediated through calcium transport protein channels or specific membrane transport proteins involved in ion transport across plasmalemma [8]. In cereal crops, Cd had adverse effects on seed germination, growth, transpiration, anti-oxidative systems, photosynthetic rate, nitrogen assimilation and yield [5,7,8].

Tak Province in northwestern Thailand is located in close proximity to the Padaeng zinc (Zn) mining site, having areas exhibiting excessive levels of Cd in agricultural soils where health and environmental problems have been identified [9,10]. This has raised strong concerns because the International Water Management Institute discovered significant Cd contamination in rice grains and paddy soils in this province [11]. These elevated Cd levels (ranging from 3.4 to 284.0 mg kg^−1^ in agricultural areas) were remarkably higher relative to the European Community limit of 3 mg kg^−1^, posing a high risk to the environment and human health [12]. The augmented deposition of Cd in water bodies and agricultural soils threatened the health status of plants, animals and humans.

Many physicochemical methods have been widely used to reduce toxicity and recover polluted agricultural sites. Alternatively, one bioremediation method using bioadsorbents, i.e., microorganisms, to reduce Cd mobility in soils could be adopted to cope with metal pollution in soils [13,14]. The adsorption/removal of environmental pollutants via soil microbial metabolic potential provides an economical and safe technique compared with other physicochemical methods. Bioremediation using indigenous heavy-metal-resistant microorganisms conferring heavy metal removal and plant-growth-promoting potential would prove a promising choice for agricultural sustainability in metal-contaminated soil.

Biofertilizers are usually formed by the (semi-)solid-state fermentation of agro-industrial wastes and consist of both beneficial microbes and primary nutrients or plant-growth-regulating substances [13,15]. Incorporating biofertilizers in soil would help produce antibiotics and stimulate biodegradation of soil organic matter (OM), thereby increasing nutrient supplies and enhancing plant tolerance to environmental stress. Microbial strains isolated from polluted environments exhibited resistance to higher levels of metals than those isolated from unpolluted areas [16]. Through metal-stress responsive mechanisms, soil microbes applied as biofertilizers effectively promoted the growth of plants implanted in heavy-metal-enriched soils by lowering metal-mediated phytotoxicity [17,18]. In addition, other mechanisms, i.e., plant-growth-promoting bacteria (PGPB), could boost plant development. For instance, they protected colonizing plants by suppressing pathogens by producing antibiotics, hydrogen cyanide and phenazines, etc. [19,20]. Additionally, PGPB could enhance plant growth via N_2_ fixation [21], solubilization of insoluble phosphorus [22], formation of siderophores [23] and phytohormones [22,24], reducing ethylene content [25], synthesizing antibiotics and antifungal metabolites and inducing systemic resistance [26]. Also, PGPB could increase soil fertility and, in turn, plant yield by providing essential nutrients and growth-regulating substances [27], lowering ethylene-based stress by inducing 1-aminocyclopropane-1-carboxylate deaminase and facilitating plant resistance to abiotic contaminants, e.g., metals and pesticides [26,28]. Previous research revealed that inoculating with plant-growth-promoting rhizobacteria (PGPR) improved maize growth and limited shoot Cd contents in the presence of Cd, as compared with the untreated control [29]. Exploiting the potential of PGPB to detoxify metals as well as versatile plant advantageous characteristics constituted a potent eco-friendly metal bioremediating tool. Hence, biofertilizers have been recognized as clean and efficient soil conditioners or amendments to improve soil characteristics [30,31].

The efficiency of biofertilizers containing indigenous Cd-resistant microbial consortia isolated from Cd-contaminated soil in reducing the phytotoxicity in rice *(Oryza sativa* L.) has not been well documented. Accordingly, soil with long-term Cd contamination caused by the mining and smelting activities in the Tak Province of Thailand was selected as our focus. The overall objective of this study was to evaluate the effect of biofertilizer amendment on alleviating Cd phytotoxicity by assessing plant growth performance, physiological response and Cd bioaccumulation within different parts of Thai rice cultivar PSL2 until the physiological maturity growth stage. The effects of test biofertilizer on soil properties and bioavailable Cd content were also examined at the growth end stage.

## 2. Results

### 2.1. Relative Abundance and Composition Structure of the Test Biofertilizers

The relative abundance and composition of the test biofertilizers BFs were assessed and compared with the enriched bacterial culture consortia BC using a 16S RNA gene amplicon sequencing approach. Bacterial composition in the phyla of the BF was summarized (Table 1). The alteration in the diversity indices of the BF was compared with the BC (Table 2). Diversity indices of the BF were significantly higher than those of the BC, as shown by the increase in the Shannon diversity index and the Chao1 richness estimator.

**Figure 1:**
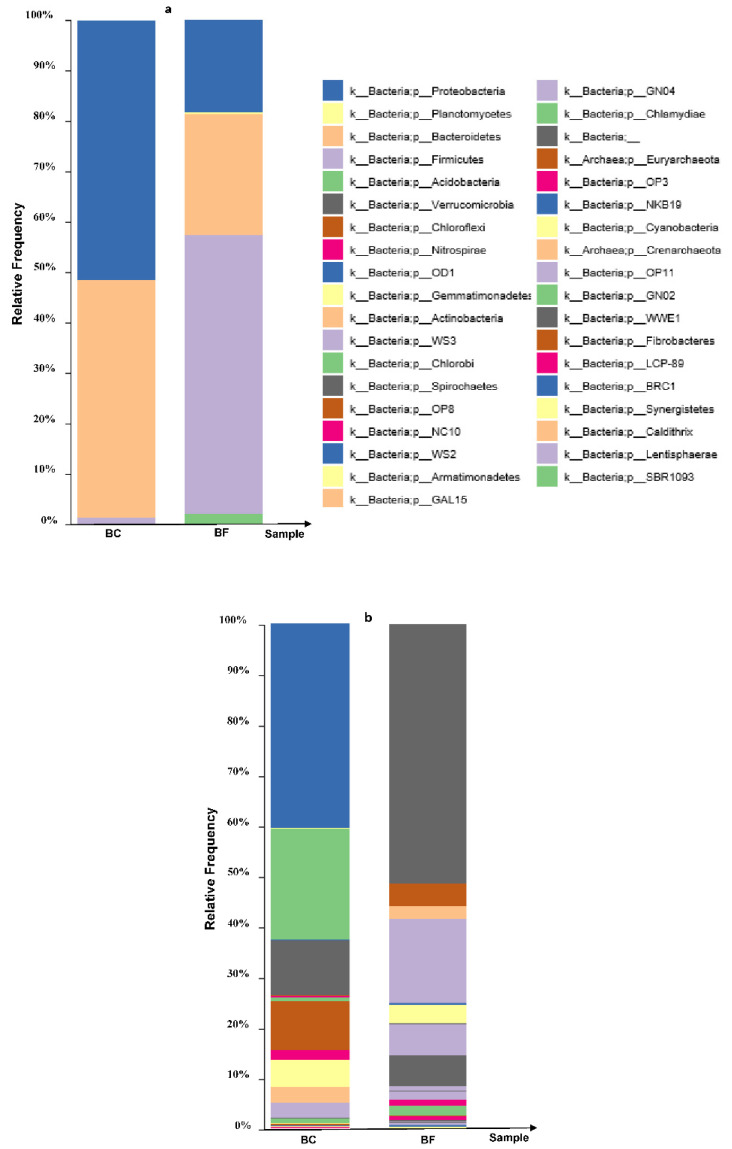
Relative abundance levels of dominant bacterial phyla (**a**) and genera (**b**) in the enriched Cd-resistant bacterial consortium (BC) (cultivable Cd) and the corresponding biofertilizer (BF) based on 16S rRNA gene Illumina MiSeq sequencing. The dominant phyla in the BF and the BC include *Proteobacteria, Bacteroidetes, Firmicutes*, *Gemmatimonadetes* and *Acidobacteria*.

The relative abundance of bacterial phyla in the BF and the BC. *Proteobacteria, Bacteroidetes, Firmicutes, Gemmatimonadetes* and *Acidobacteria* were the most dominant phyla (Figure 1). This enriched population of Cd-resistant bacteria was inoculated into the test BF used in this study. Using 16S RNA gene amplicon sequencing, the three top main phyla including *Proteobacteria, Bacteroidetes* and *Firmicutes* were explored in the BF relative to the BC (Table 2 and Figure 1a). The relative abundance of *Acidobacteria, Actinobacteria, Chloroflexi, Gemmatimonadetes, Planctomycetes* and *Verrucomicrobia* was found to be minor. Although a decrease in *Acidobacteria, Gemmatimonadetes* and *Planctomycetes* was observed, they still somewhat remained throughout the biofertilization process.

In the BF, predominant detoxifiers at a finer taxonomic level of *Proteobacteria* (including *Comamonas* sp., *Pseudomonas* sp., *Stenotrophomonas* sp., *Acinetobacter* sp., *Arcobacter* sp. and *Delftia* sp.), *Bacteroidetes* (including *Wautersiella* sp., *Myroides* sp., *Cloacibacterium* sp. and *Paludibacter* sp.) and *Firmicutes* (including *Enterococcus* sp., *Lactobacillus* sp. and *Lactococcus* sp.) were explored among other genera (Figure 1b). The biofertilizer containing the indigenous Cd-resistant bacterial consortium was successfully prepared and subjected to subsequent investigation.

### 2.2. Effect of Biofertilizer on Soil Physicochemical Traits

The biophysicochemical properties in the phyla of the biofertilizer BF relative to the organic fertilizer OF were summarized (Table 1). Under Cd stress, the BF amendment showed a maximum increase in soil pH, EC, CEC and OM as well as major minerals (N, P and K) and essential minor/trace elements (Ca and Mg) as Cd competitors (Table 3). This might be a result of the intrinsic pH and nutrients of the BF (Table 1). The amendments, by promoting soil pH (Table 3) and OM content (Table 3) and limiting tissue Cd content (Table 4), were ranked in the order of BF > OF. The increase in soil pH and OM content (Table 3) by the test amendments could lower soil bioavailable and tissue contents of Cd (Table 3 and Table 4, respectively), as compared with the control CD.

### 2.3. Effect of Biofertilizer on Rice Growth under Cd Stress

Cd exposure could impair plant growth, resulting in limited shoot and root length as well as biomass accumulation. All the amendments increased the biomass and length of rice shoot and root under Cd stress, as compared to the non-amended stressed control CD (Table 5). BF amendment in the presence of Cd showed a maximum increase in shoot and root dry weight (46% and 53%, respectively) as well as shoot and root length (27% and 45%, respectively), as compared to the control CD.

### 2.4. Effect of Biofertilizer on Mineral Homeostasis and Cd Bioaccumulation toward Cd Stress

Due to the extent of stress and growth depending on nutrient uptake and translocation, Cd toxicity caused a reduction in the major mineral (N, P and K) and minor/trace element (Ca and Mg) content of rice tissues (Table 4).

The Cd-mediated reduction in shoot N and P contents as well as shoot and root K contents were suppressed by all test amendments (Table 4). Similarly, the losses in the Ca and Mg content of the shoot and root were suppressed by the test amendments (Table 4). Among the other amendments, the BF amendment in the presence of Cd was effective in elevating the maximum levels of tissue N, P, K, Ca and Mg (Table 4).

Cd exposure increased the Cd content of rice parts, with it being much higher in the root than in the shoot (Table 4). The BF amendment in the presence of Cd remarkably reduced tissue Cd contents especially in rice grain (*p* < 0.05).

### 2.5. Effect of Biofertilizer on Photosynthetic Pigments, MDA and Proline Content in Response to Cd Stress

Cd toxicity caused a loss of photosynthetic pigments, resulting in chlorotic symptoms. Herein, Cd stress caused a remarkable decrease in chlorophyll (Chl) a, Chl b and carotenoid contents, whereas the BF amendment effectively suppressed these losses of pigments in the presence of Cd (Table 6). Among other amendments, the BF amendment showed a maximum increase in Chl a, Chl b, total Chl and carotenoid synthesis (150%, 125%, 144% and 114%, respectively), in the presence of Cd.

Malondialdehyde (MDA), a by-product of lipid peroxidation due to oxidative stress, was increased (59%) in the rice leaves under Cd stress (Table 6). Intriguingly, the BF amendment was effective in suppressing the Cd-mediated increase in MDA content.

Cd stress disturbed the water balance, leading to osmolyte accumulation in the rice leaves, as indicated by a great increase in proline content (Table 6). Under Cd toxicity, all the amendments except organic fertilizer OF prevented the accumulation of shoot proline. The BF amendment was effective in suppressing the Cd-mediated accumulation of proline by 39%.

### 2.6. The Effect of Biofertilizer on Enzymatic Antioxidant Activity upon Cd Stress

Among the enzymatic antioxidants, the activities of the antioxidant enzymes superoxide radical decomposer SOD (35%, 46%) and hydrogen peroxide scavenger APX (40%, 32%) of leaves and roots, respectively, were stimulated by Cd stress, as compared to the non-stressed control seedling CK (Table 7). In the presence of Cd, all the amendments further increased SOD and APX activity, as compared to the control CD. The BF amendment was more effective in promoting Cd-induced SOD (44%, 34%) and APX (38%, 39%) activity both in leaves and roots, respectively.

Similarly, Cd stress induced the activities of the antioxidant enzymes GPX (40%, 33%) and GR (38%, 43%) of leaves and roots, respectively. The BF amendment showed a substantial increase in the GPX (23%, 33%) and GR (23%, 36%) activity of leaves and roots, respectively, as compared to the control CD (Table 7). Among other amendments, the BF amendment showed maximum activities of SOD, APX, GPX and GR, as compared to the control CD (Table 7). By contrast, the activities of the antioxidant enzymes CAT (56%, 49%) and GST (53%, 51%) of leaves and roots, respectively, were decreased due to Cd stress, but BF amendment in the presence of Cd suppressed these losses in CAT (38%, 43%) and GST (48%, 47%) activity, compared to the control CD (Table 7). It is worth noting that the tested biofertilizer was effective in alleviating the Cd toxicity by modulating the activities of SOD, APX, GPX, GR, CAT and GST in the rice tissues with a likely strong impact on roots.

## 3. Discussion

Among metals, Cd is recognized as a highly toxic metal that impacts the growth and physiological processes, particularly during the early stages of growth and development of the plant, thereby leading to significant yield loss [5,7,8]. Exposure of rice plants to Cd stress negatively affected seed germination [32]. To reduce toxicity and remediate polluted agricultural areas, soil amendments of biofertilizer containing bioagent and fertilizer are renowned with multi-modalities to enhance plant tolerance to environmental stress, nutrient supplies and biodegradation of organic matter [13]. Indeed, indigenous microbial strains isolated from a long-term polluted paddy site displayed resistance to Cd at higher levels than those isolated from unpolluted soil [32]. The efficiency of biofertilizers derived from indigenous Cd-resistant microbial consortia isolated from Cd-contaminated soil in mitigating Cd phytotoxicity has not been well elucidated yet.

**Figure 2:**
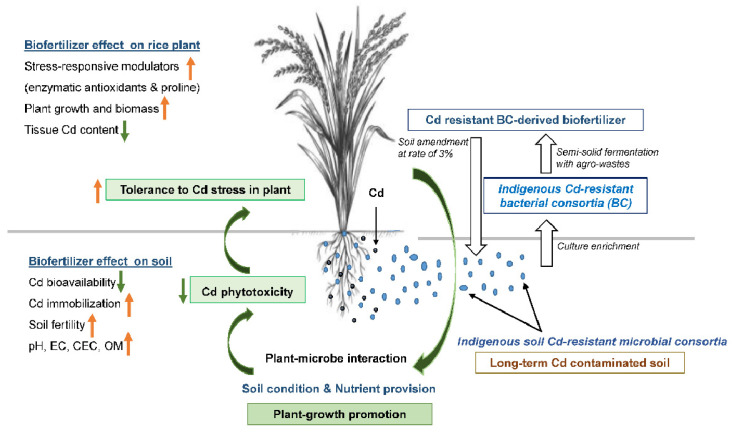
Scheme showing mitigating potential of indigenous Cd-resistant soil-microbe-derived biofertilizer to Cd toxicity. Application of biofertilizer containing indigenous Cd-resistant soil microorganisms could alleviate Cd stress in PSL2 Thai rice cultivar grown in contaminated soil, due to improved soil biophysicochemical and nutrient availability, thereby leading to lower tissue Cd content and restored growth. Especially, soil pH increased after applying biofertilizer could limit soil Cd bioavailability and toxicity to rice roots. Beneficial phyla detected in the biofertilizer, i.e., *Bacteroidetes, Firmicutes* and *Proteobacteria*, could help plants cope with Cd-induced oxidative stress by adjusting antioxidative defense, water relation and photosynthetic systems. Orange-and green-colored small arrows indicate increase and decrease of that parameters, respectively.

Our study firstly demonstrated that the soil amendment of biofertilizer containing indigenous cadmium-resistant microbial consortia could mitigate the adverse effect of Cd and improve plant growth by restoring photosynthetic pigment losses and supplying essential nutrients in Thai rice cultivars (PSL2). Additionally, the test biofertilizer consisting of bioagent and fertilizer was effective in overcoming water imbalance and oxidative stress by adjusting the content of proline and the activities of antioxidant enzymes, respectively, and limiting Cd bioaccumulation by manipulating soil physicochemical traits and mineral homeostasis (Figure 2).

In the biofertilizers, *Proteobacteria, Bacteroidetes, Firmicutes* and *Gemmatimonadetes* were determined as the most dominant bacterial phyla using 16S rRNA gene Illumina MiSeq sequencing (Figure 1). In agreement with findings from related studies, the biofertilizer pH and organic carbon could affect the abundance of *Bacteroidetes, Gemmatimonadetes* and *Proteobacteria*, and these phyla were also dominant in biofertilizer [33]. It suggested that the biofertilizer pH and organic carbon played key roles in shaping the enriched microbial communities. Moreover, soil Cd bioavailability could positively influence the microbial communities in the biofertilizer-treated soils, and some Cd-coexistence bacteria, i.e., *Chloroflexi, Acidobacteria* and *Saccharibacteria*, might have become dominant due to excess Cd in rhizosphere soils [33]. In addition, the solubility and availability of soil phosphate were determined by specific microbial activities, while soil phosphate concentration became a determinant for Cd phytotoxicity [33].

One promising method for alleviating Cd stress and promoting plant growth is the bioaugmentation of microorganisms. For instance, the application of the amendment of *Pseudomonas aeruginosa, Bacillus subtilis, Cupriavidus taiwanensis* and *Beauveria bassiana* to soil significantly restricted tissue Cd content in rice (*Oryza sativa* L.) and improved plant growth performance under Cd stress, owing to the biotransformation of the toxic Cd form to nontoxic insoluble form of Cd sulfide (CdS) and adsorption by Cd-binding proteins [34,35]. Another related study showed that a multifunctional biofertilizer containing PGPB *Pseudomonas putida* PT promoted maize growth and reduced shoot Cd contents in Cd-contaminated soils [29,36]. Very recent findings indicated that highly Cd-tolerant bacterial strains, *Pseudomonas putida* 23483 and *Bacillus* sp. GY16, had Cd removal efficiency in solution via biosorption and passivated soil via Fe-Mn binding in the rhizosphere of rice plants [37]. Related findings revealed that adding Cd-resistant *Comamonas* sp. XL8 mitigated Cd toxicity and oxidative stress in rice seedlings via intracellular formation of Cd nanoparticles [38]. Moreover, co-inoculation of *Comamonas* and *Enterobacter* species improved rice defense response, alleviated Cd stress and reduced Cd content in rice grain via Cd immobilization in soils under a pot experiment [39]. Although soil microorganisms have been extensively used to promote plant performance and restrict soil Cd, adding microorganisms into fields together with an appropriate organic substrate is recommended to maintain their substantial activities over a long period [40]. The combined advantages of bioagents and fertilizers would be an alternative to mitigate Cd phytotoxicity.

Our study revealed that the application of the biofertilizer BF increased soil chemical properties such as pH, EC, CEC and OM content (Table 3), in addition to a decrease in DTPA-extractable Cd and Zn (Table 3). Mineral nutrition containing basic cations (K, Ca and Mg) in the biofertilizer could contribute to the increase in soil pH, EC and CEC, while organic residues derived from agricultural wastes were involved in the nourishment of soil organic carbon. An increase in soil EC and CEC could also be regarded as a key factor in modulating heavy metal exchangeability and bioavailability [41,42]. Increasing soil pH after applying biofertilizer could increase the negatively charged surfaces as well as alkaline conditions, such as hydroxide and carbonate groups, which could support active substances for surface sorption, precipitation and complexation, thus reducing heavy metal bioavailability with less possibility to enter the food chain [43]. In addition, soil OM could stabilize soil Cd via microbial reduction of native oxidized soil constituents upon watering submergence [44]. OM harboring negatively charged surfaces could form stable complexes with metal ions, leading to limiting metal solubility and bioavailability to plants [45]. OM showed an effect on lowering soil Cd bioavailability and bioaccumulation in rice via Cd adsorption and the formation of stable complexes with Cd [14].

Compared to the control, the BF amendment remarkably increased soil pH, but the OF amendment had less impact on increasing soil pH (Table 3). This could be due to the initial pH of the amendments (Table 1). Although applying the OF amendment had a slight effect on soil pH, this amendment still decreased Cd contents in rice tissues compared to the control (Table 3 and Table 4). These results indicated that besides pH, the mineral nutrients, e.g., N, P, K, Ca, Mg, S and Fe, and microorganisms in the biofertilizer might play crucial roles in controlling Cd uptake and bioaccumulation [46]. Related studies reported that soil pH, DTPA-extractable Cd, total phosphorus and organic carbon were determined as the most pivotal environmental factors contributing to the changes in microbial community composition [33,47,48]. Additionally, soil organic carbon serving as the carbon source for bacteria was considered crucial. Indeed, soil pH and OM were considered important factors for limiting Cd availability [33,49]. Moreover, potential parameters other than soil pH and OM, including the mineral nutrition, e.g., N, P, K, Ca, Mg, S and Fe, and microorganisms in the biofertilizer itself remained effective in suppressing Cd bioaccumulation [14,33,50].

The retarded plant growth toward Cd stress could be due to various factors, which adversely affect water status, nutrient assimilation, mineral homeostasis, photosynthesis, oxidative stress response, etc. Our study indicated that the biofertilizer BF was effective in promoting growth performance in rice plants under Cd stress (Table 5). This might be elucidated by the fact that beneficial microorganisms, as well as mineral nutrition, in the biofertilizer could facilitate Cd immobilization in the soil and lower its toxicity to roots, possibly allowing plants to assimilate more nutrients [13,51].

Among the other amendments, the biofertilizer BF exhibited the lowest level of tissue Cd as shown by the decrease in grain Cd content, compared to the non-amended control (Table 4). This implied that the test biofertilizer had a mitigating effect on limiting Cd uptake and accumulation in rice due to the higher pH and OM content of the biofertilizer BF (Table 1 and Table 3), both of which were responsible for restricting Cd in soils by facilitating the generation of stable metalo-organo complexes at elevated pH levels [49]. Cadmium is able to form Cd hydroxide at high soil pH (>7), resulting in the promotion of Cd adsorption to soils. Indeed, rice has been regarded as a Cd-sensitive plant and an accumulator of Cd, often containing >0.1 mg Cd kg^−1^ dry biomass [52]. Indeed, the nutrients in the biofertilizer itself could also promote native microbial activity in the amended soils, thereby leading to stimulated nutrient cycling, hormone production, plant symbioses and ultimately enhanced plant tolerance to stress [13,14].

One of the adverse effects of metal stress is nutrient deficiency, as toxic metal elements compete with essential minerals for plant uptake [53]. Hence, nutrient homeostasis plays a role, in part, in preventing metal accumulation, thereby reducing their toxicity and further facilitating physiological mechanisms under Cd stress. Our study indicated that Cd stress disturbed the mineral homeostasis in rice plant, as shown by the decrease in shoot and root K, Ca and Mg content (Table 4), in agreement with other previous work [54,55,56] that has demonstrated a hermetic effect of Cd on mineral homeostasis in several plant species. Furthermore, Cd-mediated oxidative stress caused membrane damage, probably leading to reduced nutrient content in roots. Herewith, the BF amendment in the presence of Cd could restore the mineral balance, suggesting that BF-related nutrient provision could mitigate the toxic effects of Cd bioaccumulation and adjust the plant’s ability to maintain its physiological functions. Similarly, organic fertilizer amendments effectively promoted rice yield in Cd-polluted soil and reduced Cd content in rice grain [57].

One of the deleterious effects due to metal stress is the remarkable destruction of the photosynthetic apparatus. The reduction in chlorophyll biosynthesis and its content was observed in various plant species under Cd stress [55,58,59]. Our study revealed a remarkable decrease in photosynthetic pigment content in terms of the Chl (a + b) and carotenoid content in rice seedling leaves under Cd stress. These losses of pigments due to Cd toxicity resulted from the suppression of relevant enzymes, thereby leading to impaired pigment biosynthesis. In addition, the peroxidative breakdown of photosynthetic pigments and the chloroplast membrane lipid responded to abiotic stress due to excessive ROS [60]. However, these photosynthetic pigment losses could be suppressed when applying the biofertilizer (Table 6), in consistence with a previous report [61]. Alleviating Chl and carotenoid content would be linked with an increase in Cd sequestration or pigment biosynthesis and/or decrease in the breakdown of pigment complexes [62].

Similar to other xenobiotic stresses, Cd stress induces the formation of free radicals (e.g., superoxide, hydrogen peroxide) that are capable of damaging the cell membrane and biomolecules (e.g., DNA, proteins) [62]. Our study reported that a by-product of membrane lipid peroxidation MDA, a main oxidative stress indicator, was noticeably increased in response to Cd stress (Table 6). This response would be attributed to an excessive level of ROS by Cd stress. However, the BF amendment suppressed the Cd-mediated generation of MDA (Table 6).

Upon Cd stress, plants exhibited a range of secondary stress symptoms, including osmotic changes [63]. Under stress, the plants utilize crucial strategies by adjusting osmolytes to alleviate the water imbalance. For instance, biosynthesis and accumulation of proline, glycine betaine and trehalose led to osmotic adjustment of Na^+^ stress inside cells to equilibrate water balance [63,64]. Our study showed an increased proline content in rice tissues under Cd stress, but this accumulation was alleviated by the BF amendment (Table 7). The exogenous application of biofertilizer could mitigate the water imbalance in Cd-stressed rice plants by modulating the biosynthesis of proline.

Plants have vital strategies for the avoidance of Cd toxicity. For the prime line on counteracting excessive ROS and serving as cellular redox buffers, the plant’s antioxidant defense system consists of antioxidants that are enzymatic (e.g., superoxide dismutase SOD, ascorbate peroxidase APX, catalase CAT, glutathione peroxidase GPX, glutathione reductase GR and glutathione-S-transferase GST) and non-enzymatic (ascorbic acid, glutathione, tocopherol and phenolic compounds) [60,63]. Both enzymatic and non-enzymatic antioxidants work simultaneously to combat Cd-induced oxidative stress. Upon exposure to metal stress, the plant’s enzymatic antioxidant activity gradually rises with increasing metal concentration while these activities drop, and the enzymatic defense system is ultimately disrupted with extremely high metal concentration.

Recent findings indicated that APX and SOD, GR and GPX, and GST and CAT were regarded as prominent enzymatic antioxidants in the plant’s defense system for scavenging toxic O_2_.^−^ radicals and converting them to H_2_O_2_ [65]. The dramatical change in APX and SOD activity toward oxidative stress was due to overproduced O_2_.^−^ and H_2_O_2_ content, which was endured by the test biofertilizer (Table 7). Our findings were also in line with another previous study that reported remarkable changes in the activities of APX and SOD as well as other enzymatic and non-enzymatic antioxidants in plant responses to Cd stress [59]. Similarly, recent research documented an increase in the activities of potent antioxidant enzymes APX and SOD and GR and GPX in a concentration-related manner (1.0 mM and 2.0 mM CdCl_2_, respectively), as compared to the control rice seedlings (*Oryza sativa* L. cv. BRRI dhan54) [65].

In addition, this Cd-related increase in GPX and GR activity (Table 7) was responsible for scavenging H_2_O_2_ and GSSG to GSH recycling, in accordance with other previous work [66]. A substantial increase in GR and GPX activity against Cd-mediated oxidative stress was due to overproduced H_2_O_2_ and GSH content (Table 7), which was endured by the test biofertilizer, similar to previous findings [65]. Detoxifying H_2_O_2_ was mediated with the AsA-GSH cycle via the GPX/GST and GR machinery system for the conversion of GSH to GSSG for AsA recycling and the recycling of GSSG to GSH, respectively, while detoxifying xenobiotics via GST [66]. By contrast, the GST activity was reduced in the Cd-stressed seedlings (Table 7), presumably because the increased antioxidant activity hampered the overproduction of H_2_O_2_. The CAT activity was also reduced under Cd stress (Table 7), similar to another previous report [67]. However, the BF amendment suppressed the Cd-mediated reduction in CAT activity (Table 7).

In summary, the biofertilizer containing the indigenous Cd-resistant bacterial consortium therefore appeared to enhance plant growth and tolerance to Cd stress. In our investigation, the soil supplementation of the test biofertilizer at a 3% applied rate in a Thai rice cultivar (PSL2) could mitigate Cd phytotoxicity by modulating the antioxidative defense system, water and nutrient homeostasis and restricting soil Cd bioavailability. These responses most likely reflected multi-modalities of the biofertilizer harboring such microbial detoxifiers and organic residues, which could help adjust the plant’s antioxidative defense and water relation together with the improvement of soil biophysicochemical traits in coping with Cd toxicity. Furthermore, the test biofertilizer would offer the development of an eco-friendly sustainable strategy for improving plant fitness and soil quality rather than those using foreign strains, with practicability and simplicity.

## 4. Materials and Methods

### 4.1. Collection and Analysis of Soil Samples

The long-term polluted top soils (<20 cm in depth) used for greenhouse experiments were sampled from an agricultural area in Pha Dei Village, Mae Sot District, Tak Province, Thailand (N 16°40′35.9″ E 98°37′37.4″), at an altitude of 197 m. The soil at this site was tilled for either rice-corn or rice-bean crops in one cropping year. The selected physicochemical characteristics of the soil are shown in Table 4. Soil samples were divided into two main portions: one for physicochemical characterizations and the other for enriched culture following biofertilizer preparation.

Soil material was homogenized, air-dried, crushed and sieved (2 mm mesh size). The following physicochemical properties of the soil were determined: pH and electrical conductivity (EC) (1:5 soil/water suspensions) using a pH meter and an EC meter, respectively; OM content by wet oxidization and titration according to the modified Walkley–Black procedure [68]; cation exchange capacity (CEC) using 1 N ammonium chloride pH 7.0 after pretreatment to remove soluble salts [69]; total N by using the Kjeldahl method [70]; extractable P by using Bray II method [71]; and extractable K using an atomic absorption spectrophotometer (Perkin Elmer AAnalyst 200, Waltham, MA, USA) after ammonium acetate extraction at pH 7.

### 4.2. Preparation and Analysis of Cd-Resistant Biofertilizer

The biofertilizers used as amendments for remediation of Cd-contaminated soils were prepared using repeated culture enrichment of the soil Cd-resistant bacteria as previously described [32], followed by semi-solid fermentation/biofertilization conditions. Topsoil (<20 cm in depth) was collected from a long-term Cd- and Zn-contaminated agricultural area in Pha Dei Village, Mae Sot District, Tak Province (N 16° 40′ 35.9″ E 98° 37′ 37.4″), at an altitude of 197 m for culture enrichment. To enrich Cd-resistant bacteria (BC), the first 5 g of each topsoil sample was added to 95 mL of nutrient broth (NB, 0.5% peptone, 0.3% meat extract, pH7.0) containing 50 or 100 ppm Cd chloride (CdCl_2_). After two weeks of consecutive incubation at 30 °C, the bacteria were cultured on nutrient agar plates (NA, nutrient broth and 1.5% agar) supplemented with CdCl_2_ for 72 h at 30 °C. The colonies of Cd-resistant bacteria were quantified as colony-forming units per milliliter (CFU ml^−1^). The test biofertilizer (BF) was prepared under aerobic conditions, using enriched BC with rice bran supplemented with micronutrients and mineral additives to stimulate fermentation. The organic fertilizer (OF) was produced by fermenting the rice bran supplemented with micronutrients and mineral additives as aforementioned in an aerobic environment, in absence of BC. The biofertilizers were stored at 4 °C prior to use in greenhouse experiments. Hence, the treatments used in this study are listed in Table 8. The main components and bacterial compositions of each amendment are shown in Table 1.

For physicochemical analyses, properties of the biofertilizers as soil amendments or conditioners were determined: pH using a pH meter and OM content using wet oxidization and titration according to the modified Walkley–Black procedure [68]. Total contents of metal elements including Cd, Zn, Ca, Mg, S, Fe and Mn in biofertilizer samples were determined using microwave digestion and quantification using an atomic absorption spectrophotometer (Perkin Elmer AAnalyst 200, Waltham, MA, USA).

Bacterial diversity and composition of the test biofertilizers compared with the enriched consortia were analyzed using 16S rRNA gene Illumina MiSeq sequencing as previously described [32]. Total genomic DNA was extracted from 10 mL of the enriched culture and the biofertilizers (three biological replicates per treatment) using QIAamp^®^ DNA Stool Mini Kit (Qiagen, Hilden, Germany) according to the manufacturer’s instructions with some modifications. The 16S rDNA (V3-V4) bacterial primers containing the Illumina overhang adapter sequences (as underlined) 341F (5′-TCGTCGGCAGCGTCAGATGTGTATAAGAGACAGCCTACGGGNGGCWGCAG) and 805R (5′- GTCTCGTGGGCTCGGAGATGTGTATAAGAGACAGGACTACHVGGGTATCTAATCC) were used for PCR amplification [72]. The PCR mixtures (25 µL) contained 12.5 µL of 2x KAPA HiFi Hot Start Readymix (KAPA Biosystems, Wilmington, MA, USA), 5 µL of each primer (1 µmol l^−1^) and 2.5 µL of target DNA (5 ng µL^−1^). The PCR cycling conditions consisted of an initial denaturation step at 94 °C (3 min), followed by 25 cycles of 98 °C (20 s), 55 °C (30 s) and 72 °C (30 s) and a final elongation at 72 °C (5 min). The PCR products were cleaned up on AMPure XP beads (Agencourt Bioscience, Indianapolis, IN, USA). The purified amplicons (550-bp fragments) were submitted to the Omics Sciences and Bioinformatics Center (Chulalongkorn University, Bangkok, Thailand) for paired-end sequencing on the Illumina MiSeq platform. Subsequently, the purified 16S RNA gene amplicons were then indexed using 2× KAPA hot-start ready mix and 5 µL of each Nextera XT index primer in a 50 µL PCR reaction, followed by 8 to 10 cycles of PCR amplification. The PCR cycling was set as aforementioned. Next, the indexed 16S RNA gene amplicons were purified on AMPure XP beads (Agencourt Bioscience, Indianapolis, IN, USA), pooled and diluted to a final loading concentration of 4 pM. Cluster generation and 250-bp paired-end read sequencing were performed on an Illumina MiSeq using the MiSeq Reagent Kit. Amplicon sequence analysis was performed with QIIME version 1.9.0. [73]. All sequence reads were sorted based on their unique barcodes, trimmed for sequence quality and clustered at 97% identity for operational taxonomic units (OTUs). The UCHIME algorithm was used to discard chimera sequences [74].

The microbial diversity index in terms of diversity (Shannon index) and richness (Chao1 index) was subsequently computed using MOTHUR [75]. To investigate the microbial composition and diversity, the Shannon diversity index, an estimator of species richness and diversity using a natural logarithm, accounts for both abundance and evenness of the taxa present, while the Chao1 richness estimator reflects diversity from abundance data and the number of rare taxa missed from under-sampling.

### 4.3. Greenhouse Experimental Design

All the experiments involving plants adhered to the relevant ethical guidelines on plant usage. Table 1 presents the treatments used in this study. A 2000 g soil sample was crushed, sieved (2 mm mesh size) and placed in a plastic pot as previously described with some modifications [33]. Biofertilizers at a rate of 3% were mixed in long-term Cd-contaminated soil. This rate was chosen since it could contribute to optimal growth performance when varying BF concentrations (1%, 3% and 5%). Hence, NPK basal fertilizer containing 0.25 g urea kg^−1^ soil, 0.15 g KH_2_PO_4_ kg^−1^ soil and 0.04 KCl kg^−1^ soil was initially dissolved in deionized water and thoroughly mixed with the soil in each pot. Subsequently, all pots were incubated with moisture at 75% of water holding capacity for five weeks to allow the nutrients in biofertilizers to be released in the soil, as well as promote the microbes in biofertilizers to work and to functionally act toward Cd stress. Thai rice seeds (PSL2) were sterilized with 5% hydrogen peroxide (H_2_O_2_) for 5 min, rinsed with distilled water and placed in a Petri dish containing two pieces of filter paper. After germination, eight rice seedlings were transplanted in each plastic pot. The pots were arranged in a randomized complete block design with six replicates for each treatment. During rice growth, each pot was irrigated every three days with distilled water to maintain soil moisture at ca. 60 to 70% of water holding capacity. Greenhouse conditions were as follows: temperature 26 to 40 °C, 55 to 70% relative humidity, 5500 to 50,000 lx light intensity and a 12/12 h photoperiod.

After 30 days of treatment, the roots and shoots were collected separately per biological replicate and stored at 4 °C for measuring proline and malondialdehyde (MDA) content, photosynthetic pigments and different antioxidant enzymes.

Four months after transplantation, the plant samples were washed with tap water and rinsed with deionized water several times until all excess soil was removed, and then the shoots and roots were harvested. Plant materials were oven-dried at 80 °C for four days before determining weight. Soil material was collected from each pot and allowed to air-dry for five days. Soil and plant samples were subjected to chemical analyses.

### 4.4. Measurement of Total Protein Content

The total protein content of rice leaves was quantified as previously described [76]. Plant leaves (0.5 g) were ground and added with phosphate buffer. The mixture was centrifuged at 2500× *g* for 10 min. The resulting supernatant (0.1 mL) was added with distilled water to make the volume up to 1 mL. This solution was added with an equal volume of alkaline CuSO_4_ reagent and shaken for 10 min. Finally, the Follin reagent was added and then incubated for 30 min at 28 ± 2 °C. Readings were measured at 650 nm. Bovine serum albumin (BSA) was taken as a reference for the calculation of total protein contents.

### 4.5. Estimation of Photosynthetic Pigments

Photosynthetic pigments (chlorophyll (Chl) a and b and carotenoids) of rice leaves were estimated as previously mentioned [77]. Plant leaves (0.5 g) were added with 10 mL of dimethyl sulfoxide (DMSO) and then heated at 65 °C in water bath for 4 h. The supernatant was separated, and its absorbance was recorded at 663 nm and 645 nm for Chl a and Chl b, respectively, and 480 nm for carotenoids.

### 4.6. Estimation of Proline Content

Proline contents were determined by using previous protocol [78]. Rice leaves (0.5 g) were ground in 80% ethanol and then heated at 80 °C for 1 h in a water bath. After centrifugation, 0.5 mL supernatant was taken into a new test tube, added with 0.5 mL dH_2_O and 1 mL of 5% phenol and placed in an incubator for 1 h. After incubation, 2.5 mL sulfuric acid was added, and the readings were measured at 490 nm.

### 4.7. Estimation of Malondialdehyde (MDA) Content

MDA content as thiobarbituric acid (TBA) reactive substances in rice tissues was estimated by using previous protocol [79]. Initially, fresh harvested rice leaves (0.5 g) were extracted by 5% trichloroacetic acid (TCA) using a chilled mortar and pestle and then centrifuged at 11,500× *g* for 15 min. The supernatants were mixed with TBA for reaction to generate the MDA, which was subsequently measured by the absorbance difference at 532 and 600 nm and calculated using an extinction coefficient of 155 mM^−1^ cm^−1^ expressed as nmol g^−1^ FW.

### 4.8. Determination of Enzymatic Antioxidant Activities

#### 4.8.1. Enzyme Extracts

For preparing enzyme extracts, 0.5 g leaves and roots were ground in 3 mL phosphate buffer (pH 7.8) and subjected to homogenization on ice. The solution was made to 5 mL and centrifuged at 12,500× *g* for 20 min at 4 °C. The supernatant was covered with aluminum foil to avoid light exposure and stored at 4 °C for subsequent enzyme assays.

#### 4.8.2. Ascorbate Peroxidase (APX) Activity

APX (EC# 1.11.1.11) activity was quantified by examining the rate of ascorbate oxidation at a wavelength of 290 nm as the previous method [80]. The reaction mixture consisted of 50 mM phosphate buffer pH 7.0, 0.1 mM H_2_O_2_, 0.5 mM ascorbic acid and 100 µL of enzyme crude extract.

#### 4.8.3. Superoxide Dismutase (SOD) Activity

SOD (EC# 1.15.1.1) activity was subjected to assess the inhibition in the photoreduction of nitro blue tetrazolium (NBT) as previous procedure [81]. Reaction mixture was taken with 50 mM sodium phosphate buffer (pH 7.6), 0.1 mM EDTA, 50 mM sodium carbonate, 12 mM L-methionine, 50 µM NBT, 10 µM riboflavin and 100 µL of enzyme crude extract. For comparison, a set of reactions with all components except the crude extract was taken as control. To start the reactions, the reaction tubes were exposed to white light for 15 min. Reactions were terminated by switching off the lights, and readings were recorded at 560 nm.

#### 4.8.4. Glutathione Peroxidase (GPX) Activity

Glutathione peroxidase (EC# 1.11.1.9) activity was determined according to the previous procedure [82], where the reaction buffer contained 100 mM potassium phosphate buffer (pH 7.0), 1 mM EDTA, 1 mM sodium azide (NaN_3_), 0.12 mM NADPH, 2 mM GSH and 1 unit of GR. The reaction used 0.6 mM H_2_O_2_ as substrate, and the activity was calculated using extinction coefficient of 6.6 mM^−1^ cm^−1^.

#### 4.8.5. Glutathione Reductase (GR) Activity

Glutathione reductase (EC# 1.6.4.2) activity was determined according to previous protocol [83] by observing the decline in absorbance at 340 nm, where reaction mixture consisted of 100 mM potassium phosphate buffer (pH 7.0) and 1 mM EDTA. The reaction was NADPH-dependent and initiated with GSSG. Glutathione reductase activity was finally calculated using 6.2 mM^−1^ cm^−1^ as extinction coefficient.

#### 4.8.6. Glutathione S-Transferase (GST) Activity

Glutathione S-transferase (EC# 2.5.1.18) activity was spectrophotometrically examined according to previous protocol [84], where the reaction mixture contained 1.5 mM GSH, 1 mM 1-chloro-2,4-dinitrobenzene (CDNB) and enzyme crude extract. The increase in absorbance was read at 340 nm for 1 min and the enzyme activity was computed using the CDNB extinction coefficient of 9.6 mM^−1^ cm^−1^.

#### 4.8.7. Catalase (CAT) Activity

Catalase (EC# 1.11.1.6) activity was determined according to the previous method [85] by observing the decrease in absorbance at 240 nm for 1 min (as conversion of H_2_O_2_ to water and O_2_), where the enzyme extract was used to initiate the reaction. The activity of enzyme was computed using 39.4 M^−1^ cm^−1^ as extinction coefficient.

### 4.9. Sampling and Metal Analyses of Soil and Plant Tissue

Total and extractable contents of metal elements in soil and plant samples were determined using microwave digestion and diethylenetriamine pentaacetate (DTPA) extraction, respectively. Total (strong acid-extractable) Cd and Zn in soil before and after planting were estimated by digesting approximately 1 g of air-dried soil with 4.5 mL of 37% hydrochloric acid (HCl), 1.5 mL of 65% nitric acid and 1 mL of 30% H_2_O_2_ in a microwave digestion system (Milestone ETHOS One, Twinsburg, OH, USA). A similar procedure was employed to digest plant materials but without HCl addition. The amount of DTPA-extractable Cd and Zn in soil was determined using the mixture (0.005M DTPA, 0.01 M CaCl_2_ and 0.1 M triethanolamine, pH 7.30) at a soil-to-solution ratio (*w*/*v*) of 1:2. The metal concentrations in both the digests and extracts were quantified using an atomic absorption spectrophotometer (Perkin Elmer AAnalyst 200, Waltham, MA, USA).

### 4.10. Statistical Analyses

Data were subjected to statistical analysis using two-way ANOVA (SPSS Software version 18) to detect significant differences with 95% confidence level (*p*-value ≤ 0.05).

## 5. Conclusions

Amending with biofertilizer derived from indigenous Cd-resistant microbes was effective in mitigating the Cd phytotoxicity by modifying the soil biophysicochemical traits to restrict Cd bioavailability and enhance plant tolerance toward environmental stress. The promoting effect of biofertilizer could be due to a rise in soil pH, nutrient homeostasis and its enrichment of beneficial detoxifiers such as *Bacteroidetes, Firmicutes* and *Proteobacteria*, which stabilized soil Cd and limited its bioavailability, in addition to triggering stress-responsive modulators such as antioxidative enzymes and proline osmolyte. Our results indicated that the test biofertilizer as combined bioagents with fertilizer could not only nourish soil fertility but also serve as an effective amendment, especially at an applied rate to immobilize Cd in the polluted soil. These findings introduced a promising means for the sustainable development of strategies in the bioremediation of Cd-contaminated soils and plant growth improvement. Further study should be designed in the field setting to determine the efficiency of other organic residues in the heavy metal decontamination in soils to reduce the health risk of exposure to excessive toxic metals via the food chain resulting from anthropogenic environments.

## Figures and Tables

**Table 1:** Physicochemical properties and bacterial composition in phyla of biofertilizer.

Biofertilizer		BF	OF
Total viable count (CFU g^−1^)		7.8 × 10^8^	-
Bacterial composition in phyla (%)	*Acidobacteria*	1.46	-
	*Bacteriodetes*	25.89	-
	*Firmicutes*	55.45	-
	*Gemmatimonadetes*	0.12	-
	*Proteobacteria*	16.69	-
	*Planctomycetes*	0.27	-
	Others	0.12	-
pH		8.07	7.14
OM (mg kg^−1^)		4035	3046
N (mg kg^−1^)		618	814
P (mg kg^−1^)		922	611
K (mg kg^−1^)		1205	761
Ca (mg kg^−1^)		84	107
Mg (mg kg^−1^)		82	98
S (mg kg^−1^)		28	22
Fe (mg kg^−1^)		39	35
Mn (mg kg^−1^)		2.3	5.13
Zn (mg kg^−1^)		3.2	4.8
Cd (mg kg^−1^)		ND	ND

Note: pH (1:5 soil/water); CEC, cation exchange capacity; OM, organic matter; Total N, total nitrogen; Ext. P, extractable phosphorus; Ext. K, extractable potassium; Ca, calcium; Mg, magnesium; S, sulfur; Fe, iron; Mn, manganese; Zn, zinc; Cd, cadmium; ND, not detectable; BF, biofertilizer containing indigenous Cd-resistant bacterial consortium; OF, organic fertilizer as amendment control.

**Table 2:** Summary of 16S rRNA gene Illumina MiSeq sequencing data and diversity estimates for each sample.

Sample	Process	Reads	OTUs	Coverage	Chao1	Shannon
BC#1	Enrichment	60,999 ± 8307	3042 ± 198	0.998	1514.74	4.60
BC#2	Enrichment	61,629 ± 5829	3021 ± 314	0.998	1670.77	4.54
BC#3	Enrichment	61,347 ± 6018	3092 ± 268	0.997	1497.53	4.76
BF#1	Biofertilization	61,034 ± 8109	3008 ± 229	0.996	5451.82 **	10.72 **
BF#2	Biofertilization	62,182 ± 6102	3034 ± 312	0.997	5515.27 **	10.83 **
BF#3	Biofertilization	63,179 ± 7019	3063 ± 251	0.998	5672.43 **	10.92 **

Note: ** indicates respective significant difference at *p*-value ≤ 0.05, by comparing the selected parameters (Chao1 richness or Shannon diversity estimator) of the bacterial enriched consortia to those of the corresponding biofertilizers. OTUs, operational taxonomic units; BC, Cd-resistant bacterial consortia after Cd-supplemented culture enrichment of the naturally polluted topsoil samples; BF, biofertilizers after semi-solid-state fermentation process of the corresponding enriched consortium.

**Table 3:** Effect of biofertilizer containing indigenous Cd-resistant bacterial consortium on soil physicochemical properties and metal contents (N, P, K, Ca, Mg, Cd and Zn) in potting system after plantation under Cd stress.

Treatment	pH	EC (dS m^−1^)	CEC (cmol kg^−1^)	OM (%)	Total N (mg kg^−1^)	Ext. P (mg kg^−1^)	Ext. K (mg kg^−1^)	Ext. Ca (mg kg^−1^)	Ext. Mg (mg kg^−1^)	Total Cd (mg kg^−1^)	Ext. Cd (mg kg^−1^)	Total Zn (mg kg^−1^)	Ext. Zn (mg kg^−1^)
Before planting													
CD	6.65	0.27 ^b^	10.4 ^a^	1.09 ^a^	2118 ^a^	8.7 ^a^	164.3 ^a^	938.4 ± 67.4 ^a^	347.2 ± 46.2 ^a^	69.1 ± 10.3 ^a^	8.91 ± 0.22 ^c^	729.5 ± 62.3 ^a^	117.8 ± 18.6 ^c^
After planting													
CD	6.47	0.12 ^a^	9.5 ^a^	1.18 ^a^	2202 ^a^	9.4 ^a^	153.4 ^a^	923.8 ± 64.8 ^a^	338.7 ± 46.9 ^a^	68.4 ± 11.2 ^a^	8.84 ± 0.19 ^c^	718.8 ± 69.9 ^a^	108.7 ± 21.6 ^c^
OF.D	6.93	0.54 ^c^	15.8 ^b^	2.47 ^b^	6229 ^b^	19.4 ^b^	212.4 ^b^	1156.4 ± 54.3 ^b^	458.2 ± 43.5 ^b^	66.4 ± 10.5 ^a^	5.68 ± 0.21 ^b^	696.2 ± 50.2 ^a^	94.5 ± 13.5 ^b^
BC.D	6.97	0.78 ^c^	16.6 ^b^	2.38 ^b^	6083 ^b^	20.9 ^b^	206.8 ^b^	1123.7 ± 64.8 ^b^	423.3 ± 48.4 ^b^	60.4 ± 10.7 ^b^	5.17 ± 0.23 ^a^	683.8 ± 45.5 ^a^	92.3 ± 11.4 ^a^
BF.D	7.83	1.68 ^d^	19.8 ^c^	2.93 ^c^	7645 ^c^	25.8 ^c^	298.5 ^c^	1134.8 ± 52.9 ^b^	434.6 ± 32.5 ^b^	62.4 ± 8.6 ^b^	5.03 ± 0.16 ^a^	702.2 ± 53.3 ^a^	90.8 ± 12.6 ^a^

Note: All the values are the mean of three replicates ± standard error of means. Different lowercase letters indicate statistically significant difference between treatments (*p* ≤ 0.05). Details of treatments as given in Table 8. BC, Cd-resistant bacterial consortium after culture enrichment; BF, biofertilizer containing indigenous Cd-resistant bacterial consortium; OF, organic fertilizer as amendment control; pH (1:5 soil/water); EC, electrical conductivity; CEC, cation exchange capacity; OM, organic matter; Ext., extractable; N, nitrogen; P, phosphorus; K, potassium; Ca, calcium; Mg, magnesium.

**Table 4:** Effect of biofertilizer containing indigenous Cd-resistant microbial consortia on mineral nutrition (tissue N, P, K, Ca and Mg content, as expressed in mg kg^−1^) and tissue Cd content (shoot, root and panicle, as expressed in mg kg^−1^) of PSL2 Thai rice cultivar grown in Cd-enriched soils in potting system.

Treatment	Shoot N	Shoot P	Shoot K	Root K	Shoot Ca	Root Ca	Shoot Mg	Root Mg	Shoot Cd	Root Cd	Panicle Cd
CK	9.4 ± 0.8 ^b^	4.3 ± 0.6 ^b^	12.1 ± 0.9 ^b^	14.2 ± 1.2 ^d^	21.2 ± 3.8 ^b^	29.3 ± 4.2 ^c^	2.7 ± 0.9 ^c^	1.6 ± 0.5 ^c^	ND	ND	ND
CD	3.2 ± 0.7 ^a^	1.9 ± 0.4 ^a^	8.3 ± 0.6 ^a^	2.8 ± 0.5 ^a^	15.8 ± 1.7 ^a^	20.1 ± 1.7 ^a^	0.6 ± 0.2 ^a^	0.4 ± 0.1 ^a^	10.9 ± 2.1 ^c^	403.2 ± 87.6 ^a^	11.7 ± 2.4 ^d^
OF.D	18.7 ± 3.5 ^c^	8.5 ± 1.1 ^c^	14.2 ± 1.5 ^c^	4.1 ± 0.7 ^b^	24.1 ± 2.2 ^c^	25.4 ± 2.5 ^b^	1.2 ± 0.5 ^b^	0.6 ± 0.3 ^b^	8.6 ± 1.6 ^b^	412.9 ± 63.5 ^a^	6.9 ± 2.6 ^c^
BC.D	21.5 + 1.3 ^c^	10.9 ± 1.4 ^c^	15.6 + 2.3 ^c^	4.8 + 1.1 ^b^	25.6 + 1.4 ^c^	26.7 + 3.3 ^b^	1.5 + 0.3 ^b^	0.8 + 0.2 ^b^	7.3 ± 1.8 ^a^	424.6 + 81.3 ^a^	5.4 ± 1.9 ^b^
BF.D	24.9 ± 3.1 ^d^	12.9 ± 1.8 ^d^	16.4 ± 3.7 ^c^	5.6 ± 1.3 ^c^	26.8 ± 2.3 ^c^	27.6 ± 1.9 ^b^	1.9 ± 0.7 ^b^	1.0 ± 0.3 ^b^	6.8 ± 1.1 ^a^	435.9 ± 71.6 ^a^	3.6 ± 1.7 ^a^

Note: All the values are the mean of three replicates ± standard error of means. Different lowercase letters indicate statistically significant difference between treatments (*p* ≤ 0.05). Details of treatments as given in Table 8. BC, Cd-resistant bacterial consortium after culture enrichment; BF, biofertilizer containing indigenous Cd-resistant bacterial consortium; OF, organic fertilizer as amendment control; N, nitrogen; P, phosphorus; K, potassium; Ca, calcium; Mg, magnesium; ND, not detectable.

**Table 5:** Effect of biofertilizer containing indigenous Cd-resistant microbial consortia on biomass and length of shoot and roots of PSL2 Thai rice cultivar grown in Cd-enriched soils in potting system over 4 months.

Treatment	Shoot DW (mg Plant^−1^)	Root DW (mg Plant^−1^)	Shoot Length (cm on Average)	Root Length (cm on Average)
CK	149.3 ± 1.8 ^c^	76.4 ± 4.8 ^c^	85.2 ± 7.0 ^c^	49.1 ± 8.4 ^c^
CD	78.2 ± 3.8 ^a^	35.1 ± 2.8 ^a^	59.3 ± 5.8 ^a^	24.6 ± 4.8 ^a^
OF.D	127.4 ± 5.2 ^b^	58.7 ± 1.7 ^b^	79.2 ± 5.2 ^b^	42.5 ± 4.3 ^b^
BC.D	138.3 ± 7.1 ^c^	64.5 ± 3.5 ^c^	77.6 ± 5.5 ^b^	40.9 ± 6.1 ^b^
BF.D	145.7 ± 8.2 ^c^	75.3 ± 1.4 ^c^	81.7 ± 4.8 ^c^	45.1 ± 5.3 ^c^

Note: All the values are the mean of three replicates ± standard error of means. Different lowercase letters indicate statistically significant difference between treatments (*p* ≤ 0.05). Details of treatments as given in Table 8. BC, Cd-resistant bacterial consortium after culture enrichment; BF, biofertilizer containing indigenous Cd-resistant bacterial consortium; OF, organic fertilizer as amendment control; DW, dry weight.

**Table 6:** Effect of biofertilizer containing indigenous Cd-resistant microbial consortia on photosynthetic pigments (chlorophyll a and b and carotenoid content), MDA and proline content of PSL2 Thai rice cultivar grown in Cd-enriched soils in potting system.

Treatment	Chl a (mg g^−1^ FW)	Chl b (mg g^−1^ FW)	Total Chl (mg g^−1^ FW)	Carotenoid (mg g^−1^ FW)	MDA Content (nmol g^−1^ FW)	Proline Content (µg g^−1^ FW)
CK	1.6 ± 0.3 ^d^	1.0 ± 0.1 ^c^	2.6 ± 0.3 ^d^	1.8 ± 0.5 ^d^	14.7 ± 2.1 ^a^	0.7 ± 0.1 ^a^
CD	0.6 ± 0.2 ^a^	0.4 ± 0.1 ^a^	1.0 ± 0.2 ^a^	0.7 ± 0.2 ^a^	35.7 ± 4.3 ^d^	3.6 ± 0.2 ^e^
OF.D	1.0 ± 0.5 ^b^	0.6 ± 0.2 ^b^	1.6 ± 0.4 ^b^	1.1 ± 0.3 ^b^	27.8 ± 3.8 ^c^	2.8 ± 0.1 ^d^
BC.D	1.2 ± 0.2 ^c^	0.8 ± 0.1 ^c^	2.0 ± 0.2 ^c^	1.3 + 0.2 ^c^	22.1 ± 2.7 ^b^	1.9 ± 0.2 ^c^
BF.D	1.5 ± 0.8 ^d^	0.9 ± 0.2 ^c^	2.4 ± 0.7 ^d^	1.5± 0.3 ^d^	19.6 ± 3.5 ^b^	1.4 ± 0.3 ^b^

Note: All the values are the mean of three replicates ± standard error of means. Different lowercase letters indicate statistically significant difference between treatments (*p* ≤ 0.05). Details of treatments as given in Table 8. BC, Cd-resistant bacterial consortium after culture enrichment; BF, biofertilizer containing indigenous Cd-resistant bacterial consortium; OF, organic fertilizer as amendment control; Chl, chlorophyll; Total Chl = Chl (a + b); MDA, malondialdehyde; FW, fresh weight.

**Table 7:** Effect of biofertilizer containing indigenous Cd-resistant microbial consortia on enzymatic antioxidant activities (SOD, APX, GPX, GR, GST and CAT, as expressed in EU mg^−1^ protein) of PSL2 Thai rice seedling grown in Cd-enriched soils in potting system.

Treatment	Leaf SOD	Root SOD	Leaf APX	Root APX	Leaf GPX	Root GPX	Leaf GR	Root GR	Leaf CAT	Root CAT	Leaf GST	Root GST
CK	24.1 ± 3.4 ^a^	26.3 ± 1.8 ^a^	62.9 ± 8.4 ^a^	84.3 ± 3.8 ^a^	60.2 ± 8.4 ^a^	80.4 ± 3.8 ^a^	20.4 ± 3.4 ^a^	25.6 ± 1.8 ^a^	48.4 ± 3.4 ^c^	40.2 ± 1.8 ^b^	67.2 ± 8.4 ^c^	83.8 ± 3.8 ^b^
CD	37.2 ± 5.8 ^b^	48.4 ± 3.8 ^b^	105.3 ± 7.6 ^b^	124.3 ± 12.1 ^b^	100.5 ± 7.6 ^b^	120.4 ± 12.1 ^b^	32.7 ± 5.8 ^b^	44.8 ± 3.8 ^b^	21.3 ± 5.8 ^a^	20.7 ± 3.8 ^a^	31.5 ± 7.6 ^a^	41.2 ± 12.1 ^a^
OF.D	40.6 ± 6.1 ^b^	52.4 ± 5.2 ^b^	116.5 ± 8.6 ^b^	135.8 ± 11.6 ^b^	110.6 ± 8.6 ^b^	130.5 ± 11.6 ^b^	34.6 ± 6.1 ^b^	50.2 ± 5.2 ^c^	30.5 ± 6.1 ^b^	32.6 ± 5.2 ^b^	51.1 ± 8.6 ^b^	68.3 ± 11.6 ^b^
BC.D	58.4 ± 7.9 ^c^	67.4 ± 7.1 ^c^	142.6 + 9.1 ^c^	187.2 ± 11.8 ^c^	134.2 + 9.1 ^c^	157.8 ± 11.8 ^c^	38.5 ± 7.9 ^c^	60.7 ± 7.1 ^d^	32.8 ± 7.9 ^b^	34.7 ± 7.1 ^b^	57.4 + 9.1 ^c^	74.8 ± 11.8 ^b^
BF.D	66.2 ± 7.3 ^d^	73.7 ± 8.2 ^c^	169.4 ± 17.1 ^d^	202.4 ± 10.1 ^c^	156.9 ± 17.1 ^d^	180.2 ± 10.1 ^d^	42.6 ± 7.3 ^c^	70.3 ± 8.2 ^e^	34.5 ± 7.3 ^b^	36.3 ± 8.2 ^b^	60.7 ± 17.1 ^c^	78.2 ± 10.1 ^b^

Note: All values are the mean of three replicates ± standard error of means. Different lowercase letters indicate statistically significant difference between treatments (*p* ≤ 0.05). Details of treatments as given in Table 8. BC, Cd-resistant bacterial consortium after culture enrichment; BF, biofertilizer containing indigenous Cd-resistant bacterial consortium; OF, organic fertilizer as amendment control. FW, fresh weight; EU, enzymatic unit; SOD, superoxide dismutase; APX, ascorbate peroxidase; GPX, glutathione peroxidase; GR, glutathione reductase; CAT, catalase; GST, glutathione S transferase.

**Table 8:** Experimental treatments and their nomenclature used in this study.

Symbol	Treatments
CK	Non-amended plants under normal condition
CD	Non-amended plants under Cd stress
OF.D	Plants amended with organic fertilizer (OF) under Cd stress
BC.D	Plants inoculated with indigenous Cd-resistant bacterial consortium (BC) under Cd stress
BF.D	Plants amended with biofertilizer (BF) containing indigenous Cd-resistant bacterial consortium under Cd stress

## Data Availability

All data generated or analyzed during this study are included in this published article and information files.
